# Interventions to Support System-level Implementation of Health Promoting Schools: A Scoping Review

**DOI:** 10.3390/ijerph13020200

**Published:** 2016-02-06

**Authors:** Jessie-Lee D. McIsaac, Kimberley J. Hernandez, Sara F.L. Kirk, Janet A. Curran

**Affiliations:** 1 Atlantic Health Promotion Research Centre, Dalhousie University, Halifax, NS B3H 4R2, Canada; Sara.Kirk@dal.ca; 2 Interdisciplinary Studies, Dalhousie University, Halifax, NS B3H 4R2, Canada; Kimberley.Hernandez@dal.ca; 3 School of Nursing, Dalhousie University, Halifax, NS B3H 4R2, Canada; jacurran@dal.ca

**Keywords:** health promoting schools, health promotion, school interventions, organizational innovation, implementation science, systems

## Abstract

Health promoting schools (HPS) is recognized globally as a multifaceted approach that can support health behaviours. There is increasing clarity around factors that influence HPS at a school level but limited synthesized knowledge on the broader system-level elements that may impact local implementation barriers and support uptake of a HPS approach. This study comprised a scoping review to identify, summarise and disseminate the range of research to support the uptake of a HPS approach across school systems. Two reviewers screened and extracted data according to inclusion/exclusion criteria. Relevant studies were identified using a multi-phased approach including searching electronic bibliographic databases of peer reviewed literature, hand-searching reference lists and article recommendations from experts. In total, 41 articles met the inclusion criteria for the review, representing studies across nine international school systems. Overall, studies described policies that provided high-level direction and resources within school jurisdictions to support implementation of a HPS approach. Various multifaceted organizational and professional interventions were identified, including strategies to enable and restructure school environments through education, training, modelling and incentives. A systematic realist review of the literature may be warranted to identify the types of intervention that work best for whom, in what circumstance to create healthier schools and students.

## 1. Introduction

School health interventions are most effective in supporting chronic disease and cancer prevention when they target multiple environmental and behavioural components and incorporate a complementary community strategy [1,2,3,4]. Health promoting schools (HPS) is recognized globally as a multifaceted approach that can support prevention behaviours of healthy eating, physical activity and tobacco use by modifying the social and physical environments in schools [5,6]. In doing so, HPS advocates for health promotion (HP) actions that influence social norms and improve access, resources, and supports for healthy behaviours [5,6]. There are increasing international calls to action that advocate for increased support for HPS [7]. However, this support may be undermined by persisting challenges that obstruct uptake of HPS across multiple levels [8,9,10,11,12]. Although a recent review identified factors facilitating a HPS approach at a school-level [13], there is limited synthesized knowledge on the broader elements that facilitate a HPS approach at a school system-level. 

Systems-thinking offers a theoretical lens that recognizes interrelationships and interactions across stakeholders that are tied together by organizational structures, processes and contexts but require stronger linkages to promote behaviour change within and across systems [14,15,16]. This perspective is appropriate for interventions within the education system considering the interdependency of a range of system-level elements [17] (e.g., government ministries may develop HPS standards and offer professional development for groups of schools). Bronfenbrenner’s ecological model offers an overall framework to consider the interrelationship among actors and structures across multiple levels in schools; the *macrosystem* influences the overall institutional structure, such as the educational, social, economic and political systems; the *exosystem* constitutes other informal and formal social structures, such as government ministries or departments and school districts that are responsible for a sub-section of schools; *microsystems* focus on relationship between individuals and their immediate settings; and *mesosystems* comprise interactions between *microsystems* [18]. Implementation of a HPS approach aligns with an ecological model as it is influenced by education and social policy (*macrosystem*) and by social structures (*exosystem*) that can shape the availability of resources to help build supportive physical and social environments (*microsystem*) [5,19,20,21,22,23]. HPS is also a collaborative and multi-component approach that engages partners in the community (*mesosystem*), such as public health, recreation, non-government organizations, local business and universities [5]. Focusing on system-level actions can ensure synergy between decisions (*macro-* and *exosystem*) and operations [24] (*microsystem*).

Although there is increasing clarity around factors that enable HPS to operate successfully at a school level (e.g., school leadership, culture and capacity within the school, support from parents/community) [13], there is limited synthesized knowledge on the broader system-level elements that facilitate a HPS approach. Examining the capacity of the overall system is important as it allows a consideration of how actors work together to engage in the creation and co-production of interventions to improve performance, adaptation and innovation related to a HPS approach [23,24,25,26,27]. For example, different levels of the system could implement actions that focus on alignment of curriculum with HPS goals, professional development and networking opportunities that encourage knowledge exchange and provision of resources to support implementation. These actions may address local implementation barriers and ensure synergy between decisions and operations across education systems to support uptake of HP evidence [24]. Michie and colleagues considered behaviour change as part of an interacting system and recently validated the Behaviour Change Wheel (BCW) as a method of characterizing and designing behaviour change interventions for tobacco control and obesity prevention [28]. This comprehensive framework was developed from a review of 19 behaviour change frameworks and provides a useful structure to characterize the types of interventions and policies needed to support system-level uptake of a HPS approach [28]. The BCW links policy options and intervention functions to sources of behaviour that could be targeted to achieve change. Intervention functions are described as nine broad categories of interventions that could be used to change behaviour. Seven policy categories are detailed that support delivery of the different intervention functions (see Table 1) [28].

Scoping review studies offer an approach to knowledge synthesis that broadly describe relevant literature across various study designs. Scoping reviews are conducted to examine the range of existing literature and gaps related to a topic, to determine the need for a systematic review or to summarise and disseminate research findings [29,30]. Considering the importance of a systems-level perspective on a HPS approach and the lack of synthesized literature on system-level elements, a scoping review was warranted to identify, summarise and disseminate the range of research to support the uptake of a HPS approach across school systems.

**Table 1:** Definitions and examples of intervention functions and policy categories with examples from this review relevant for a HPS approach (adapted from Michie and colleagues [28]).

Policies	Definitions	Examples
Communication/marketing	Using print, electronic, telephonic or broadcast media	Not described explicitly but may have included national campaigns to raise awareness
Guidelines	Creating documents that recommend or mandate practice. This includes all changes to service provision	Standards for HPS
Fiscal	Using the tax system to reduce or increase the financial cost	Not described
Regulation	Establishing rules or principles of behaviour or practice	Inspections of HPS integration at schools
Legislation	Making or changing laws	Integration of HPS into school mandates and curriculum
Environmental/social planning	Designing and/or controlling the physical or social environment	Supporting implementation and changing environmental norms
Service provision	Delivering a service	Training courses, management or coordination of HPS
**Intervention function**	**Definition**	**Example**
Education	Increasing knowledge or understanding	Information sharing related to HPS.
Persuasion	Using communication to induce positive or negative feelings or stimulate action	Not described
Incentivisation	Creating expectation of reward	Reward or recognition for HPS; integration of HPS within school/educator responsibilities and
Coercion	Creating expectation of punishment or cost	Not described
Training	Imparting skills	Workshop and other training sessions to teach key skills
Environmental restructuring	Changing the physical or social context	Encouraging implementation of HPS actions to change context
Modelling	Providing an example for people to aspire to or imitate	Networking, sharing andn exchange opportunities for schools
Enablement	Increasing means/reducing barriers to increase capability or opportunity	Providing support and resources to facilitate HPS implementation

The second purpose was to determine existing gaps in the literature of the review to determine the need for a more systematic review of the literature. We used Bronfenbrenner’s ecological model to consider systems-level influences as well as the BCW policy categories and intervention functions to inform potential strategies to influence HP policy and practice [28]. The review responds to the following question: What intervention functions and policy categories support implementation of a HPS approach across school systems?

## 2. Methods

We adhered to Arksey and O’Malley’s [29] guidelines on scoping reviews and the recommendations outlined by Levac and colleagues [30] to maximize the rigour and utility of our review findings. The details of the methods are outlined in Table 2 according to the six framework stages. The population for this review included various types of *school systems* (e.g., collections of schools within a country, province/state, local district/board rather than individual schools) related to student health outcomes focused on modifiable risk factors for chronic disease and cancer (e.g., physical activity, healthy eating, tobacco control). Within the scope of this literature, the focus was to identify the types of system-level interventions that can facilitate uptake of a HPS approach at a school-level.

The primary author worked in collaboration with a health sciences librarian to develop a search strategy including relevant search terms for three electronic bibliographic databases of peer-reviewed literature: PubMed, Web of Science, ERIC (Years 1999–2015, see Table 2). The primary author also consulted with a group of 14 international HPS experts to identify other important studies to include in the review. Two reviewers independently screened titles using pre-specified inclusion/exclusion criteria to determine their suitability for the review. The reviewers discussed discrepancies, challenges and uncertainties related to study selection after independent review. During subsequent screening, remaining abstracts and then full-text articles were independently reviewed for inclusion by the same two reviewers. The reference lists of included studies were further hand-searched to identify additional relevant articles and provide further system and jurisdictional context related to the included articles. Data were extracted from included studies by two reviewers using a structured data extraction sheet (see Table 3). Key findings across all studies were synthesized by coding extracted data according to the variables of interest to identify implications for research, policy and practice [30].

**Table 2:** Scoping review framework stage details.

Framework Stage	Details
Step 1: Identifying research question	**Scoping review question:** What are the various intervention functions and policy categories that support implementation of HPS across school systems? **Target population:** School system (not individual schools). **Health outcomes:** Modifiable risk factors for chronic disease and cancer (e.g., physical activity, healthy eating, tobacco control).
Step 2: Identifying relevant studies	**Search concepts:** Health Promoting Schools; knowledge translation/ implementation science; and systems strategies. **Search terms:** (school OR education setting *; OR school health services and health promotion OR Health Policy OR Health promoting school * OR comprehensive school health OR coordinated school health) and (Implement OR implements OR implementing OR implementation * OR knowledge development * OR knowledge translation OR knowledge utilization * OR knowledge utilisation * OR knowledge exchange * OR information dissemination) and (capacity building OR partnership OR collaboration OR cooperative Behavior * OR cooperative behaviour * OR program development OR program planning OR evidence-based Practice * OR decision-making OR organizational innovation).
Step 3: Study selection	**Inclusion criteria:** Publication time scan (1999–2015, date based on release of HPS reviews in 1999); English language; school system actions (not individual schools); intervention/primary study published in peer-reviewed paper. **Exclusion criteria:** Not focused on actions at the system-level (*i.e.*, focus on individual schools or beyond school/education system); health outcomes not focused on modifiable risk factors for chronic disease (physical activity, healthy eating, tobacco control) and mental health; concept paper without information on a specific education system.
Step 4: Charting the data	Two reviewers extracted data from final articles and charted according to variables of interest, including policy categories and intervention functions.
Step 5: Collating, summarising and reporting the results	Results synthesized and interpreted according to the research questions as well as key issues and themes relevant for practice and policy.
Step 6: Consultation	Results will inform future consultation with experts and identification of next steps, including possible inclusion of grey literature and/or systematic realist review.

* indicates that variations on the word was searched.

## 3. Results

**Figure 1:**
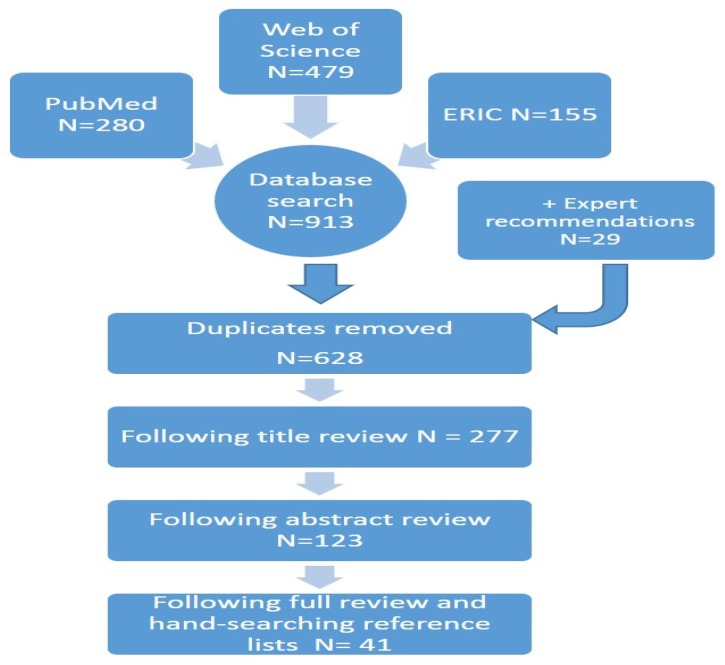
Scoping review flow chart.

A total of 628 studies were identified for possible inclusion, reduced to 277 after title review and 123 after abstract review (Figure 1). Through an iterative process of full article review and hand searching of reference lists between two reviewers, 41 articles met the final criteria for the review. Table 3 describes the final studies included as part of this review. Studies were grouped according to similar system-level context (*i.e.*, multiple studies about the same regional school system or project). Overall, the included studies targeted all levels of the school system in Austria, Norway, United Kingdom, Canada (provinces Alberta, Quebec and the Youth Excel network), United States (including states Michigan, New England, Pennsylvania, Colorado, Arizona, South Carolina), Australia (Western Australia and South Australia ), New Zealand and the Western Pacific (Taiwan and Hong Kong). The majority focused on the school system across both primary and secondary schools (*n* = 31, with seven of these also including other school types such as vocational and preschool), including some with younger children (e.g., pre-school) and others with post-secondary school (e.g., vocational, technical and vocational schools. Other systems focused solely on secondary schools (*n* = 3) or did not specify the age of students in the article (*n* = 7). Various research methods were used across all studies, including descriptive program review, case study methodology and qualitative and quantitative methods.

### 3.1. Ecological System Context 

Included studies are described below according to the ecological system context in their respective region and country and their relevance to the ecological system (ecological level identified in *italicised* font).

**Table 3:** Overall study results (HPS = health promoting schools; HP = health promotion).

Authors, Year Reference	Population	Study Aim/Objective	Study Type, Design, Participants	Intervention Function (Based on Individual Studies or Related Groups of Studies Where Identified)	Policy Categories (Compiled Across studies According to Country/Region)
Flaschberger, Nitsch, & Waldherr, 2012, [34].	Unspecified	To assess compatibility and connectivity through a HP pilot training course.	Qualitative: focus groups. Teachers and principals (*n* = 37).	**Training**: course on HPS implementation; **Education**: shared HPS information; **Incentivisation**: tools and money for HPS actions; **Modeling**: cooperative online learning phase.	**Austria Guidelines:** quality standards of HPS and establishment of health management in schools; **Service provision:** teacher training, health management in schools.
Gugglberger, 2011 [33].	Unspecified	To investigate capacity-building measures for HP and develop a typology of support strategies.	Qualitative: interviews. Reps from provincial organizations (*n* = 23).	**Enablement**: coordination of HP actors, information, consultation and information (also education); **Environmental restructuring (ER)**: changing culture of school to meet HPS goals; **Modelling**: exchange among schools.	
Gugglberger & Duer, 2011 [32].	Primary, secondary and other school types **^1^**	To investigate capacity building to facilitate the implementation of the HPS concept.	Qualitative: interviews. Principals (*n* = 11).	**Enablement**: capacity building measures important for HPS improvements (also incentivisation, modeling, education and training); **ER**: identified factors influencing institutionalization of HPS.	
Gugglberger, Flaschberger, & Teutsch, 2014 [36].	Unspecified	To investigate the possible side effects of HP in schools.	Qualitative: group discussions. Teachers (*n* = 63) and head teachers (*n* = 9) acting as network coordinators.	**Education/Training**: opportunities for teachers to gain new skills, **ER**: improved infrastructure for to HPS, **Enablement**: required to reduce negative side effects (also incentivisation).	
Flaschberger, Gugglberger, & Dietscher, 2013 [35].	Primary, secondary and other school types	To investigate how different stakeholders of network schools perceive learning.	Qualitative: interviews (*n* = 9) and group discussions (*n* = 17) with network steering group and HPS coordinators.	**Enablement**: provisions of means to support HP; **Training**: workshops, on-site coaching, **Education**: lectures, seminars; **Modelling**: learning from network schools; **ER**: implementation of HP with network support.	
Samdal, Viig, & Wold, 2010 [40].	Primary and secondary schools	To identify prerequisites for implementation of HP in a HPS network.	Mixed methods: school documents and materials, teacher interviews (*n* = 12) from 2 schools.	**Norwegian Network of HPS Enablement**: funding for programming; **ER**: integrating HPS programs into daily work of the school; **Training**: seminars including all school nurses and HPS coordinators; **Incentivisation**: resources for HPS, **Modelling**: professional learning through sharing and competence building across HPS schools (also education).	**Norway Environmental/social planning**: implementation of HPS programming for three years; **Guidelines**: HPS implementation based on baseline studies; **Service provision**: annual training, school health coordinator.
Viig, Fosse, Samdal, & Wold, 2012 [37].	Primary and secondary schools	To examine how program leaders in a HPS Network managed, facilitated, and supported HP initiatives.	Qualitative: focus group with program leaders (*n* = 10).		
Tjomsland, Iversen, & Wold, 2009 [38].	Primary and secondary schools	To examine teacher motivation, participation and outcomes in a HPS network.	Quantitative: teacher surveys at baseline and after participation in the network (*n* = 104)		
Viig, Tjomsland, & Wold, 2010 [41].	Primary and secondary schools	To investigate conditions related to teacher participation in a HPS network.	Quantitative: teacher surveys. Baseline and after participation in the network (*n* = 104)		
Viig & Wold, 2005 [39].	Primary and secondary schools	To examine teachers’ perception of school factors that facilitated participation in HP.	Qualitative: interviews with teachers from 2 network schools (*n* = 12).	Enablement: compensating teachers for time spent on HP (also incentivisation); **Modelling**: networks with other teachers; **ER**: focus on school planning documents.	
Gugglberger & Inchley, 2012 [42].	Primary, secondary schools	To analyse the processes that led to school HP implementation.	Qualitative case study: interviews with actors at national, local and school levels (*n* = 14).	**Enablement**: support from national leadership, development of strategic vision and award criteria; **ER**: embedding HP actions into the school system through curriculum and award system (also incentivisation).	**Scotland Legislation**: HPS integrated into schools by law through curriculum; **Regulation**: regular health and well-being inspections; **Environmental/Social Planning**: changing the health and wellness norms at all schools; **Guidelines**: national HPS standards; **Service provision**: training opportunities.
Inchley, Currie, & Young, 2000 [44].	Primary and secondary schools	To investigate the processes with adoption of HP and identify key stages in becoming a HPS.	Descriptive: report of multiple case study approach including process and outcome evaluation.	**Enablement**: coordinator with time and resources; **Modelling**: multi-agency steering groups, links between primary and secondary schools; **Training**: offered for school staff and administration; **Education**: building awareness of HP to community.	
Inchley, Muldoon, & Currie, 2007 [43].	Primary and secondary schools	To explore the processes involved in developing and implementing the HP at local level.	Descriptive: report on process evaluation of multiple case study approach and focus group with students and teachers.	**Enablement:** financial resources to support HP development (also incentivisation); **Training**: provided from external professionals (also education); **ER**: improving the physical environment of the school.	
Rothwell, Shepherd, Murphy, Burgess, Townsend & Pimm, 2010 [45].	Primary and secondary schools	To assess the implementation of a network of Healthy School Schemes at national, local and school levels.	Qualitative case study: documentary analysis, interviews with coordinators (*n* = 23) and stakeholder discussion at three workshops.	**Enablement** through training and funding (also incentivisation) and other supports; **ER**: focus on implementing HPS to modify school environment; **Modelling**: clusters of school staff meet and discuss; **Training**: coordinators transfer skills to school staff.	**Wales, United Kingdom Guidelines**: Assessment schemes. **Environmental/ Social planning**: Focused on planning and implementing prioritized activities; **Service provision**: coordinators support schools.
Gleddie, 2012 [52].	Primary and secondary schools	To examine the effectiveness of a local school district implementation model of the HPS.	Qualitative case study of school district project: focus groups of teacher champions and principals, interviews with teachers and principals; document review and personal observations and interactions (participant #s not specified).	**Battle River HPS Project, Alberta Enablement**: implementation support, sub-coverage and professional development (also incentivisation, education and training); **ER**: embedding health into procedures, actions and frameworks informed by reports (also education and enablement); **Modelling**: sharing evidence and exchange across schools.	**Canada Guidelines**: framework for HPS; **Environmental/social planning**: each provincial/territorial jurisdictions provides different support for implementation.
Gleddie, 2012 [51].	Primary and secondary schools	To examine the development and implementationof a comprehensive healthy school policy as part of HPS implementation.			
Gleddie & Hobin, 2011 [53].	Primary and secondary schools	To describe how evidence was used to support changes in the school environment and health behaviours.	Descriptive: context and evidence collected during case study		
Murnaghan, Morrison, Griffith, Bell, Duffley, McGarry & Manske, 2013 [48].	Primary and secondary schools	To describe lessons learned from a tri-provincial case study of knowledge exchange systems for youth health.	Qualitative case study: document analyses, interviews (*n* = 90), focus groups (*n* = 15) and online survey.	**Youth Excel network, Various provinces/territories Enablement**: exchange opportunities helped explain stakeholder roles and facilitate action planning and use of common indicators across schools (also education); **Modelling**: Networks, coalitions and working relationships; **ER**: focus on using evidence to inform action.	
Riley, Wong, & Manske 2014 [49].	Primary and secondary schools	To evaluate the how knowledge development and exchange capacity was built.	Qualitative: interviews with network members (12 researchers, 5 practitioners and 4 policy makers).		
Riley, Manske, & Cameron, 2011 [47].	Primary and secondary schools	To provide rationale and describe progress for vision to jointly set HP actions.	Descriptive: program review and description of model.		
Deschesnes, Drouin, & Couturier, 2013 [55].	Secondary schools	To describe a conceptual framework that identifies core features likely to facilitate the incorporation of HPS into school functioning.	Descriptive: literature review, 2 case studies including interviews, document review and observations (participant #s not specified).	**Quebec Enablement**: framework supported conceptualization of HPS, provision of technical support for implementation (also environmental restructuring) and opportunities for sharing (also modelling); **Education**: model reinforced knowledge and understanding of principles of HPS and professional development for schools (also training).	
Deschesnes, Tessier, Couturier, & Martin, 2013 [56].	Primary and secondary schools	To document the professional development model implementation process and assess the influence on knowledge and practices about HP.	Qualitative case study: document analysis, interviews with principals and facilitators (*n* = 12), meeting observations with 3 schools.		
Deschesnes, Couturier, Laberge, & Campeau, 2010 [57].	Unspecified	To examine stakeholders’ conceptions of HP and provide understanding to stakeholders’ positions on HPS dissemination.	Mixed methods: document analysis, interviews with key informants at provincial, regional and local levels (*n* = 34).		
Mcbride, 1999 [68].	Primary and secondary schools	To present the project model for creating HP in schools based on the experience of working with schools.	Descriptive: model and program description.	**Western Australian School Health Project Enablement**: providing resources and supports (also training, education and incentivisation) to support HP planning (also environmental restructuring) improved comprehensiveness and quality of health strategic planning.	**Australia Guidelines**: framework for HPS; **Environmental/social planning**: Each jurisdiction provides different support for implementation.
Mcbride, 2000 [69].	Primary and secondary schools	To evaluate changes in school HP practice related to low and high intensity intervention (mail-out resources *vs.* training, planning time and expert support).	Quasi-experimental research: matched comparison schools (*n* = 11 for each group). Interviews and observations at 3 time points to assess HP support and activity.		
Rana & Alvaro, 2010 [70].	Primary and secondary schools	To assess the effectiveness of using a HPS framework to deliver nutrition intervention in schools.	Mixed methods evaluation: program attendance, workshop feedback, menu assessment, case studies, feedback and interviews (*n* = 68 schools)	**Enablement**: providing information, workshops (also education and training) and grants (also incentivisation); **Modelling**: sharing between schools; **ER**: focus on planning and implementing HPS approach.	
Lynagh, Knight, Schofield, & Paras, 1999 [71].	Secondary schools	To provide an overview of intervention model based on HPS and trialed in a pilot study and the reported barriers.	Descriptive: describes randomized control trial with junior secondary schools (*n* = 22)	**Enablement**: liaison officer supported HPS adoption (also environmental restructuring); **Education**: newsletters, resources, reports, **Training**: workshops with some sharing between schools (modelling).	
Cass, Price, & Rimes, 2005 [72].	Primary and secondary schools	To investigate a school HP grants scheme as a strategy to support HPS.	Descriptive: program review including quantitative data and qualitative interviews (participant #s not specified).	**Enablement**: offering grants to support HPS-related programming in schools (also environmental restructuring and incentivisation)	
Liu, Chang, Liao, Niu, Cheng, Shih, Chang & Chou, 2015 [74].	Primary and secondary schools	To examine the impact of expanding school–district/university partnership programs on improvement for HPS.	Quantitative: surveys with schools before and after the expanded support for HPS (*n* = 647 in 2011 and *n* = 1195 in 2013).	**Enablement**: support from university partners (also training), funding for HPS (also incentivisation); **ER**: focus on implementing action research had a positive impact on increasing HPS implementation, perceived HPS impact and efficacy (change over time and significantly higher among intervention schools).	**Taiwan Regulation**: requirement by schools to participate in HPS; **Guidelines**: guiding HPS documents. **Environmental/Social planning**: actions to facilitate HP action. **Service provision**: workshops and support from experts.
Chang, Liu, Liao, Niu, Cheng, Chou & Chang, 2014 [75].	Primary and secondary schools	To examine if an HPS action-research approach was effective in advancing HP implementation, perceived impact and efficacy in Taiwan.	Quantitative: surveys with action research (*n* = 138) and non-action research schools (*n* = 483).		
Lee, Leger, & Cheng, 2007 [79].	Primary, secondary and other school types	To report findings from baseline assessment of the status of schools participating in a Heathy Schools Award	Mixed methods: Quantitative surveys, qualitative observations and focus groups with staff and students (*n* = 98 schools).	**Enablement**: framework developed to explore what schools do for HP (also education); **Incentivisation**: awards offered based on level of HPS implementation; **ER**: focus on enhancing physical and social environment; schools that had implemented HP more comprehensively were deemed to have a healthier environment to support student health and learning.	**Hong Kong Environmental/Social planning**: Actions to facilitate action related to HPS. **Guidelines**: Standards developed for Awards Scheme.
Lee, Cheng, Fung, & St Leger, 2006 [78].	Primary, secondary and other school types	To examine differences in students’ health and learning from schools that had obtained a Heathy Schools Award.	Quantitative: surveys before and after applying for award (*n* = 9 schools).		
Lee, St Leger, & Moon, 2005 [77].	Primary, secondary and other school types	To describe the evaluation framework and data collection to school performance in aHeathy Schools Award scheme.	Quantitative: surveys (*n* = 15 schools).		
Lee, Cheng, & St Leger, 2005 [76].	Primary, secondary and other school types	To identify an evaluation framework developed for HPS.	Descriptive: evaluation framework, protocol and data collection instruments.		
Barnes, Lohrmann, Shipley, & O’Neill, 2013 [65].	Unspecified	To assess HP partnership capacity through a Coordinated School Health Leadership Institute.	Mixed methods: surveys and structured interviews (*n* = 14 participating teams).	**Enablement**: Supported HPS partnerships through financial and material resources, technical assistance and opportunities for sharing (also incentivisation, education, training and modelling); **ER**: focus on local school district activities and actions.	**United States Environmental/Social planning**: actions to facilitate action related to HPS; **Service provision**: various supports across states to facilitate HPS, including School Health Index (assessment and planning tool); **Guidelines**: national guidelines for coordinated school health (*i.e.*, HPS); **Legislation**: requirement for wellness policy if participating in national school lunch program.
Dewitt, Lohrmann, O’Neill, & Clark, 2011 [66].	Unspecified	To detect and document successes and challenges of participants in the Leadership Institute.	Qualitative: interviews from team members (*n* = 11).		
Butler, Fryer, Reed, & Thomas, 2011 [62].	Secondary schools	To describe challenges and opportunities from university/school district collaboration for HP.	Descriptive: program review.	**Enablement**: financial and human resources, technical support, opportunities for reciprocal learning between universities and schools (also incentivisation, education, modelling); **ER**: through HP planning process.	
Austin, Fung, Cohen-Bearak, Wardle, & Cheung, 2006 [60].	Primary, secondary and other school types	To examine school HP teams' experiences working with a School Health Index (SHI) and HP efforts.	Qualitative: interviews with faculty, staff and community collaborators (*n* = 34, 9 schools) after SHI and 1 year following.	**Enablement**: use of self-assessment and outside facilitators increased HPS actions. **ER**: through the use of planning data with the tool.	
Staten, Teufel-Shone, Steinfelt, Ortega, Halverson, Flores & Lebowitz, 2005 [61].	Unspecified	To describe a model that used the SHI and assistance to improve HP through case studies of implementation.	Program description: 13 school from 5 districts.	**Enablement**: support from external coordinator and financial award upon completion of SHI (also incentivisation). **ER**: changes to school environments following SHI. **Training**: external coordinators e aimed to transfer knowledge to internal coordinators.	
Valois & Hoyle, 2000 [63].	Primary and secondary schools	To describe the extent to which a HP infrastructure was in place and functioning.	Program evaluation: performance assessment of HPS model across 7 schools.	**Enablement**: resources for coordination and liaising, workshop and retreat settings focused on program development and implementation (also environmental restructuring, education, training)	
Hoyle, Samek, & Valois, 2008 [64].	Primary and secondary schools	To examine the efforts of a school district to develop, improve, and sustain HP.	Descriptive: case study	**Enablement**: internal and external supports and resources supported implementation (also incentivisation, environmental restructuring); **Incentivisisation**: supportive policies, procedures and management structures; **Training**: ongoing professional development (also education).	

**^1^** Other school types = (technical and vocational).

#### 3.1.1. Europe

There is a rich history of a HPS approach in Europe, with the European Network for HPS being established in 1993 and re-launched as “Schools for Health in Europe” to link national programmes and networks that had started to develop [31]. The network encourages its 43 member countries to develop and implement a national policy on school HP, to build on the experiences within each country [31] (*macrosystem*). In Austria, schools (*microsystem*) are dependent on varying external and political supporting structures for implementation across various provinces and regions (*exosystem*) [32]. Three Austrian studies investigated capacity building in relation to provincial support strategies [33], perceived support by schools [32] and provision of a pilot training course [34] (*micro- and exosystem*) and two other studies examined a regional network through stakeholders perceptions of learning [35] and side effects of HP [36] (*micro- and mesosystem*). Five studies in Norway discussed participation of schools in the Norwegian network of HP in terms of program leader support [37], teacher motivation and participation [38] and school implementation [39,40,41] (*micro- and mesosystem*). In Scotland, HP is well integrated into the school system, with all schools being required (and being monitored) to become a HPS since 2007 (*macrosystem*). Three studies discussed the overall national approach to support HPS, including one that focuses on the higher-level processes that led to HP implementation [42] and two earlier studies (*i.e.*, before the HPS requirement) that reported on factors involved with implementation at a school level [43,44] (*micro- and exosystem*). One study in Wales assessed the implementation of a network of healthy school schemes [45] that were established following funding that was provided to all local authorities for the appointment of healthy schools coordinators whose role was to establish and maintain local schemes (*micro-, meso- and exosystem*).

#### 3.1.2. North America

There is no federal policy related to HPS in Canada (*macrosystem*) but provinces/territories have developed and implemented guidelines and support for HPS relevant for their jurisdictional context (*exosystem*) [46]. The Pan-Canadian Joint Consortium for School Health is a partnership between provincial, territorial and federal governments in Canada that provides leadership by enhancing alignment between health and education across multiple sectors rather than creating national policy [19]. Youth Excel (Youth Health Collaborative: “Excelerating” EVIDENCE-informed ACTION) was a pan-Canadian network that aimed to accelerate the dissemination and implementation of evidence-informed HP policy and practice through collaborations across jurisdictions and research, policy and practice sectors [47]. One study provided rationale for the vision of Youth Excel to have priorities for action jointly set and actions leading to continuous improvement in HPS and described the initial progress of the network [47] (*meso- and exosystem*). Two studies described key lessons learned [48] and how knowledge development and exchange was built through the network [49] (*mesosystem*). Included studies also described the specific jurisdictional systems in two regions (*exosystem*). Ever Active Schools is a provincial non-government organization that works with school communities in Alberta to implement the HPS approach [50] (*exosystem*). The Battle River Project in Alberta was supported by Ever Active Schools and the Battle River school division and provided school with evaluation and planning tools, release time for teachers, knowledge exchange opportunities, access to expert advice and support from a part-time coordinator to implement HPS. One study described the process that led to the development of implementation of the HPS model [51], a second examined the effectiveness of the model [52] and the final discussed how evidence was used to support school implementation [53] (*micro- and exosystem*). In Quebec, a joint initiative for HP has been offered on a voluntary basis since 2004 by the Ministry of Education and the Ministry of Health and Social Services and support is provided to schools by designated regional and local agents [54]. Three related studies described a conceptual model for HPS identifying key facilitating features [55], documented a professional development model and implementation process and its influence on stakeholder knowledge and practices [56] and examined how stakeholders concepts of HP may influence dissemination across the province [57] (*micro- and exosystem*).

The United States Centers for Disease Control and Prevention supports a Coordinated School Health (similar to HPS) model and created the School Health Index (SHI) in 2000 to provide educators with a tool to help school staff evaluate the strengths and weaknesses of school-based health promotion programs and policies and plan for further improvement [58] (*marcosystem*). Further, all schools participating in the National School Lunch Program or other child nutrition programs must have created a local school wellness policy by 2006 [59] (*macrosystem*). In response to the federal system, many states and school districts have developed regional infrastructure to support implementation in schools. This review identified a study in New England and Arizona that examined the use of the SHI with support from outside community partners [60,61] (*micro-, meso- and exosystem*). The Pittsburgh (Pennsylvania) school district used the SHI planning tool for developing collaborations and solidifying relationships between a public university and the school district to help the district launch HPS [62] (*micro-, meso- and exosystem*). Two studies described case studies supporting HP through the Mariner project in South Carolina [63] and a school district in Colorado [64] (*micro- and exosystem*). Finally, the MICHIANA HPS Leadership Institute was developed to build local HP infrastructure capacity in Michigan through training that emphasizes school and community partnerships to develop HP infrastructure and provides assistance to support local school district activities and actions. One study assessed the extent to which school systems met expectations for building HP capacity [65] and another examined common themes by participants at the Institute [66] (*micro-, meso- and exosystem*). 

#### 3.1.3. Australia

In Australia, there is no federal policy specifically related to HPS and the investment by the government remains largely focused on the health sector to address specific priorities [67] (*macrosystem*). Studies identified in this review have reported on specific regional initiatives. In Western Australia, two studies reported on an intervention of over 70 schools provided ongoing support for health promotion through staff and parent training, access to an expert and time for school representatives to plan and implement health promotion activity [68,69] (*micro- and exosystem*). With South Australia, one study examined the “CREATE healthy eating in schools” program is helping to support schools to implement healthy eating guidelines in schools using a HPS approach by providing grants, workshops, resources and planning tools, training program, membership to network and nutritionist support [70] (*micro-, meso- and exosystem*). In the Hunter Region HPS model in New South Wales each school was allocated a liaison officer whose role it was to encourage adoption of HP and facilitate progression [71] (*micro- and exosystem*). Finally, in the same region the School Health Incentive Program grants scheme offers small grants, support for experts and access to services and resources was evaluated by one study in terms of its ability to enhance the capacity of schools to develop and implement health initiatives using the HPS framework [72] (*micro- and exosystem*). 

#### 3.1.4. Western Pacific

A HPS approach has been supported by the World Health Organization as part of the dissemination of their Global School Health Initiative in 1995 across the Western Pacific [73] (*macrosystem*). The Taiwan Ministry of Education and Department of Health signed a Joint Declaration of HPS in 2002 and by 2008 all primary and middle schools were required to implement HPS. In 2004, supports were initiated to facilitate HP adoption through a network, teacher training and resource center and website and support was expanded in 2011 with an action research school–district/university partnership program. Experts from universities participated in support network by providing technical support and local school action research training workshops to build teacher capacity and support implementation [74]. One study examined the support of the partnership program on continuous improvement for HP [74] and another assessed if the action research approach was effective in advancing HP implementation [75] (*micro-, meso- and exosystem*). In Hong Kong, HPS was first launched in 1998, and further developed as a Healthy Schools Award Scheme in 2001 with an aim to promote educational achievements, and to enhance the well-being of school students and staff [76]. An overall evaluation framework and data collection plan was described [76] as well as individuals studies reporting how schools performed under the award scheme [77], if comprehensive adoption of the HPS model led to improved health and learning outcomes of students [78] and the status of HPS across schools [79] (*micro- and exosystem*).

### 3.2. Policy Categories and Intervention Functions (Identified through Italicised Font)

The level of detailed description of policy context varied across included studies (Table 3). *Guidelines, environmental/social planning* and *service provision* were the most commonly reported policy categories (Table 3). Some form of *guidelines* for HP existed in all countries and many were used to facilitate implementation of HPS and change educational norms for schools (*i.e.*, environmental/social planning). *Service provision* was identified through training and coordination support. Forms of *legislation* and *regulation* were only identified in a few countries. Scotland had a legislative requirement for HPS to be integration in all schools (which was also accompanied by regular health and well-being inspections) [42], the United States has a legislative responsibility for all schools to have a wellness policy if they are participating in the national school lunch program [59] and Taiwan requires all schools to implement HPS [75]. *Communication/marketing* and *fiscal* policies were not explicitly described. 

The most common intervention functions described by authors were enablement and environmental restructuring (Table 3). Various system-level resources and infrastructure supports were mentioned as being important to support *enablement* at the local level. These included personnel resources, structures, frameworks, tailored support with quality control, information and knowledge, financial resources and opportunities for collaboration and exchange [32,36]. *Enablement* was often combined with other intervention functions including *environmental restructuring, education, training, modelling and incentivisation*. The intervention functions *persuasion* and *coercion* were not identified in this review. Various studies discussed the importance of *enablement* through leadership between health and educational sectors to achieve a negotiated and shared vision for HPS as a joint initiative [32,45,57], tailored support with evidence and planning tools [47,48,62,69,72] and flexibility that allows for development based on the local needs of schools [32,42,68,69,72] *(environmental restructuring)*. It was perceived that a balance between these conditions, that appreciates the need for “rigidity and flexibility” [51], would encourage ownership by schools to facilitate HP into practice and enhance sustainability [34,42,43,52,56,72]. Many studies also discussed the important role of a trained, effective coordinator to *enable* local planning groups to plan, implement and integrate HP [39,40,43,52,56,63,68,70]. External coordinators were perceived as being able to work across boundaries to garner support through partnerships, while also ensuring local progress and supporting evidence-based action as part of the school team through regular visits [37,45,61,71] (*enablement*). Various evidence and planning tools were used to support changes to the physical and social context of the school (*enablement, education and environmental restructuring*). For example, the SHI in the United States was used as a planning tool that facilitated collaboration and action planning for HP [61,62]. Other studies in Canada discussed the development of local surveillance systems that generated tailored products and reports provided opportunities for reflection, dialogue and action planning [49,53] and advocacy to accelerate investment [47] (*enablement*). In some cases, studies mentioned that a planning tool helped to embed HP policy and practice within the culture of the school system [53] (*environmental restructuring*). One study noted that there is a need for these tools to produce rapid results to identify strengths and areas of improvement [65]. Studies also commented on the importance of access to information to support HP and workshops/training sessions (*education and training*) to build knowledge and understanding of a HPS approach and their skills and confidence to take action [32,34,40,56,70]. 

Integration of a HPS approach within the core function of the school system was an important theme across the included studies as it *incentivised* schools to participate [52,76]. Ensuring the vision and aims of HPS were embedded with school mandates and responsibilities made it easier for teachers to participate [37] and helped to institutionalize HPS practices to ensure sustainability [32,34] and embed HP within accountability frameworks [36,51]. The compatibility of HPS with the education system was discussed in many studies as being an important *incentive* for schools and some studies mentioned this occurring through new national curricula [39,40,42] and policies [66]. If alignment was not achieved, studies reported many undesirable ‘side-effects’ [36] with professed lack of time and the busy school workload being a common barrier reported when HP was not well integrated into the system [36,39,41,61,66,71]. Studies also reported on the different types of *incentives* that were provided to encourage school participation, including financial resources through grants and provision of time for health promotion work through substitute coverage [32,34,37,39,41,51,52,61,66,71,72,74,76]. However, it is important to note that, one study mentioned that although having sufficient time was challenging, schools found ways to compensate [39]. Another study corroborated this finding by suggesting there was more of an emphasis on establishing partnerships rather than external support that may address the issue of time [40]. Collaboration and teamwork were also identified as being critical to facilitate the translation and integration of HP policy and practice [32,34,43,48,52,57,61,62,65,68,70,75,79]. Barnes and colleagues (2013) noted the importance of partnerships in helping to build organizational capacity to sustain the HPS approach [65]. 

Through collaboration, opportunities for exchange and learning with and from other schools (*modelling*) was discussed as being an opportunity to share best practices and *enable* HP [35,37,39,40,45,70]. Viig and colleagues suggested that rather than explicit training offered as a stand-alone session, ongoing professional learning was positively supported through providing opportunities for sharing and competence building within and across schools [37] (*training and modelling*). However, there were challenges to supporting this type of exchange, such as lack of resources [39] and collaboration, coordination and communication [35]. Engagement in and success of exchange opportunities in HPS generally may also be influenced by individual factors, such as teacher motivation, professional learning and discretion, or personal interest [38,41]. However, some studies suggest that, HP implementation levels, perceived HP impact and HPS efficacy of teachers are higher when there is increasing participation and system support [74,75]. 

## 4. Discussion

This review identified a range of articles describing interventions to support the uptake of the HPS approach across school systems. Bronfenbrenner’s ecological model provided overall system context across multiple levels of influence [18] and the BCW provided a useful framework to describe the different intervention functions that have been used to implement a HPS approach [28]. There were various *macrosystem* influences across the studies that corresponded with the respective country or region. Many interventions were described at the *exosystem* level to provide different types of support for *microsystem*s and some intervened to support interactions between *microsystem*s (*i.e.*, *mesosystem*). Service provision, guidelines and environmental/social planning were the most commonly represented BCW policy categories across the school systems included in this review. These policies provided high-level direction and resources within school jurisdictions that help to enable HP implementation through changes to schools social and physical environments. Communication/marketing strategies were not explicitly discussed in articles but there may have been national campaigns to promote and raise awareness of HPS that were not described. Fiscal policies were also not identified but considering that few countries reported legislative requirements, it is unlikely that any were using the tax system to reduce or increase costs. At the intervention function level of the BCW, there was a great deal of interaction described across elements. For example, although the most common main elements were enablement and environmental restructuring these were achieved in part by additional intervention functions including education, training, modelling and incentivisation. Environmental restructuring is consistent with the process of implementing a HPS approach as it requires a change to the physical and social context based on individual needs of school communities [5]. 

### 4.1. Ecological System Context

An ecological perspective supports the interpretation of the results and understanding how to support a HPS approach at a system-level. Previous studies have acknowledged that schools are social complex adaptive systems, considering their diversity and dynamic nature, nested system structure (*i.e.*, various levels of jurisdiction and clusters), dependence and autonomy and the presence of rules, feedback loops and interaction [17,22,80]. Acceptance of this perspective assumes that interventions in schools require an appreciation of the interdependence between simultaneous and mutually interactive components and suggests that interventions may not always lead to predictable change within schools [17,22]. Rather, actions may results in non-linearity and emergence as education sector policies evolve and schools respond to the interaction between various factors [17,22,33]. Therefore, although different interventions may be needed to accommodate the unique contexts of schools, having adequate, appropriate and flexible support within the school system may enable schools to make autonomous decisions and implement HP practices based on their capacity and circumstances. 

Contextual guidance has been described in the governance of complex social systems [81] and may provide a helpful strategy to consider support for HP adoption and implementation [33,42] as it combines internal self-organization (*i.e.*, local decisions and control) and external strategic framing of options (*i.e.*, providing a framework and resources). For example, providing the necessary resources and frameworks may help to ensure that schools have the support they need while fostering autonomy to implement according to their unique needs [42]. This perspective also highlights the importance of having HPS guidelines and supports available for schools while also moderating regulation as it may interfere with decision making and self-organization at the school-level [33,82]. The concept of linking and connecting schools to support modelling and exchange of diverse perspectives was also an important finding from this review [35]. Developing a learning organization and building a deeper understanding of the systemic change process can be critically important in achieving change [17]. Creating meaningful and relevant opportunities that facilitate collective visioning for alignment of a HPS approach with priorities of the education system may also help to engage all stakeholders in the change process to continuously learn and improve [17]. Evolving mindsets about education may help to shift perceptions of a HPS approach so that it is more fully integrated into the culture of schools [17,52]. Further research is needed to examine how to support organizational learning and foster understanding of change as it relates to a HPS approach.

### 4.2. Behaviour Change Wheel Components

This review identified potential interactions across the policy categories and intervention functions that were described in studies. Moderating regulation through a focus on enablement and supporting ownership of HP was an emphasis for many studies [34,42,43,51,52,56], whereas the BCW components of persuasion and coercion were not identified. This seems logical considering school ownership has been perceived as being critical to HPS sustainability [83]. The results from this review suggest that policy actions that develop HPS structures, matched with resources that build school-level action, can help to facilitate commitment to the HPS approach (e.g., through personnel resources, frameworks, tailored support with quality control, information and knowledge, financial resources and opportunities for collaboration and exchange). This is particularly important for facilitating HP actions in schools as many educators are trained to teach academic outcomes [6,84] and lack of guidance and resources for schools can limit their ability to embrace an HPS approach [34].

### 4.3. Strengths and Limitations

There are several strengths and limitations that should be noted. This review sought to identify the range of research on interventions designed to support the uptake of a HPS approach across school systems using scoping review methodology. This inclusive approach is a strength when considering such a complex topic. The two primary authors screened all potential articles according to the inclusion/exclusion criteria that were shaped using an iterative process. Key HPS content experts were consulted to identify relevant articles for the review. The focus was on peer-reviewed articles, and since a reasonable number of articles were identified, the results provide an important first step in scoping out the current evidence. Qualitative methods were used in the majority of included studies and although this provided depth to understanding potential implications of system supports, the impact of interventions were not always clear as few studies quantitatively evaluated the outcomes of system-level support. This is a limitation of the review as it precluded the ability to make direct comparisons across literature. Although evaluating impact was not the purpose of this review, it is important to note that several studies that included intervention outcomes found positive difference among changes to curriculum, social environment [38] and student well-being [78]. This scoping review also provides a first step in understanding how theoretical frameworks, such as the BCW and ecological model, may help to guide further interventions across HPS systems. An ecological model provides a framework to describe different system levels [18] and the BCW provides a mechanism to characterize interventions and match intervention types to the behavioural target, the target population and the context in which the intervention will be delivered [28]. For the BCW, there was limited information on the rationale for systems-level action (*i.e.*, if decisions were politically motivated and/or based in tacit or empirical evidence) so it was unclear if they were designed with a formal process of mapping potential policies or interventions to have specific impacts on the schools. Future research could further apply this framework to explore how HPS interventions influence the behaviour system (capability, motivation, opportunity) of schools [28]. The results from this review also form the basis of further consultation with experts on the overall findings and identification of next steps, including the inclusion of grey literature to complement this review of peer reviewed studies. A systematic realist review of the literature is warranted to identify the types of intervention that work best for whom, in what circumstance, and how, to create healthier schools and students [85].

## 5. Conclusions

This review summarises research on interventions to support the uptake of a HPS approach across school systems. Despite international advocacy to support a HPS approach, there are persisting challenges that obstruct uptake of HP actions in school systems. Existing policies provide high-level direction and resources within school jurisdictions to support HP implementation. Various multifaceted organizational and professional interventions, including strategies to enable and restructure school environments through education, training, modelling and incentives were identified as important. Strategies that incentivised schools to implement HP were particularly relevant, considering the core responsibilities of schools are typically focused on academic rather than health outcomes. Top-down (*macrosystem*) system policies, sufficient support through interventions that enable school action (*microsystem*) through resources, information, planning supports and opportunities for sharing are required (*exo- and mesosystem*). Further review is needed to determine the combination of approaches that may help to redefine current challenges and establish an imperative for schools to promote health as a precursor to achieve improved learning outcomes, advance HP implementation and creating healthier schools and students.
